# A Fatal Case of Concurrent Disseminated Tuberculosis, Pneumocystis Pneumonia, and Bacterial Septic Shock in a Patient with Rheumatoid Arthritis Receiving Methotrexate, Glucocorticoid, and Tocilizumab: An Autopsy Report

**DOI:** 10.1155/2021/7842049

**Published:** 2021-09-07

**Authors:** Shin-Ichiro Ohmura, Ryuhei Ishihara, Ayaka Mitsui, Yoshiro Otsuki, Toshiaki Miyamoto

**Affiliations:** ^1^Department of Rheumatology, Seirei Hamamatsu General Hospital, Shizuoka, Hamamatsu, Japan; ^2^Department of Pathology, Seirei Hamamatsu General Hospital, Shizuoka, Hamamatsu, Japan

## Abstract

Recently, treatment for rheumatoid arthritis has dramatically improved but increases the risk of bacterial and opportunistic infections. Herein, we report a fatal case of concurrent disseminated tuberculosis, pneumocystis pneumonia, and septic shock due to pyelonephritis caused by extended-spectrum *β*-lactamase-producing *Escherichia coli* in a patient with rheumatoid arthritis who received methotrexate, glucocorticoid, and tocilizumab. Despite undergoing intensive treatment, the patient developed respiratory failure and died after 7 days of admission. An autopsy indicated that pulmonary tuberculosis were the ultimate causes of death, while pyelonephritis was controlled.

## 1. Introduction

Rheumatoid arthritis (RA) is an autoimmune disease characterized by synovitis and structural damage to multiple joints. Treatment for RA has dramatically improved after the introduction of biologics. If patients do not show a good response during methotrexate (MTX) therapy, then rheumatologists administer biologics, including inhibitors of tumor necrosis factor *α*, interleukin- (IL-) 6, and interactions between T cells and antigen-presenting cells by blocking cytotoxic T lymphocyte-associated protein 4 and Janus kinase in combination with MTX. However, these drugs increase the risk of bacterial and opportunistic infections, including tuberculosis (TB), cytomegalovirus infection, herpes zoster infection, and *Pneumocystis jirovecii* pneumonia (PCP) [1–4].

Patients with human immunodeficiency virus infection (HIV) with CD4 count below 200/*μ*L experience opportunistic infections [5]. However, there are no surrogate markers to identify the development of opportunistic infections in non-HIV patients, including patients with RA.

Here, we report a case of a 79-year-old Japanese woman with RA who developed disseminated TB, PCP, and bacterial septic shock during MTX, glucocorticoid, and tocilizumab (TCZ) therapy.

## 2. Case Presentation

**Figure 1:**
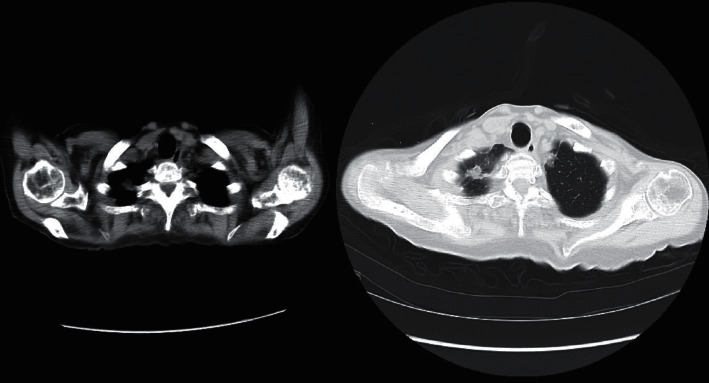
Chest CT showing thickening of the pleura and a nodule at the apex of the left lobe.

A 79-year-old woman with RA was referred to our hospital. She was diagnosed with RA because of polyarthritis and positive C-reactive protein (CRP) and anticyclic citrullinated peptide antibody. She had been receiving treatment with MTX and glucocorticoid for 1 year, although her disease activity could not be controlled with these drugs. Consequently, we initiated 40 mg of adalimumab (ADA) for every 2 weeks. However, her disease activity did not improve despite ADA treatment with 7 weeks, and 162 mg of TCZ every two weeks was initiated in combination with 12 mg of MTX every week and 12 mg of methylprednisolone per day. Before the initiation of TCZ, she did not have a history of TB or come in contact with any patient with TB. Blood tests showed a leukocyte count of 12820/*μ*L, lymphocyte count of 1615/*μ*L, CRP of 5.4 mg/dL, and immunoglobulin G (IgG) level of 686 mg/dL (Table 1). The T-SPOT-TB test is a type of T cells recognizing antigens specific to *Mycobacterium tuberculosis*, and serum *β*-D glucan was negative. She had no family history of TB. Chest computed tomography (CT) showed thickening of the pleura and a nodule at the apex of the left lobe (Figure 1). We did not administer anti-TB drugs because the T-SPOT-TB test and sputum test for TB were negative. Since we suspected her to have sulfamethoxazole/trimethoprim (SMX/TMP) allergy, 300 mg of inhaled pentamidine was administered every 4 weeks, as prophylaxis for PCP. However, her disease activity did not improve and TCZ was shortened every week. After every week TCZ treatment, her serum CRP level was 0.01 mg/dL within 1 month. However, the serum IgG levels gradually decreased, and lymphocyte count decreased on day 44 (Table 1). Two months after switching to TCZ, the patient was admitted to our hospital due to sudden high fever with a two-week history of generalized fatigue and cough. She was not in contact with anyone with an infection or coronavirus disease 2019, and she did not go to any infected areas. She was under treatment with 8 mg of methylprednisolone per day, 12 mg of MTX every week, and 162 mg of TCZ every week for RA before admission.

**Figure 2:**
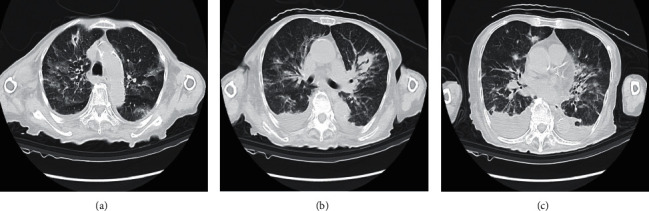
Chest CT image at the upper lobe level (a), at the carina level (b), and at the lower lobe level (c) on admission. Chest CT showed grand glass opacity in all fields with consolidation in the right and left lungs.

**Figure 3:**
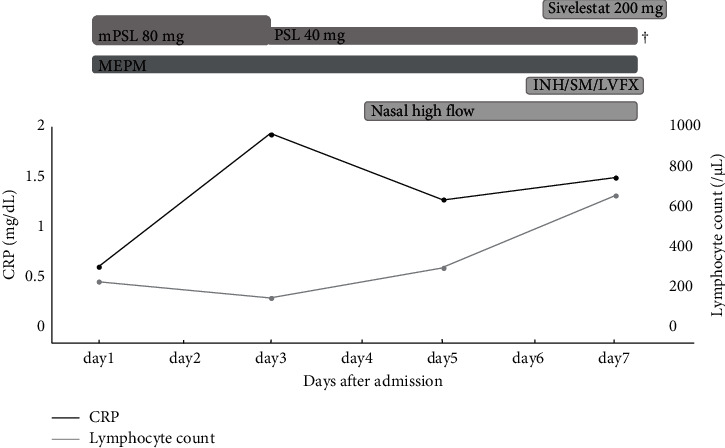
The clinical course. CRP, C-reactive protein; INH, isoniazid; LVFX, levofloxacin; MEPM; meropenem; mPSL, methylprednisolone; PSL, prednisolone; SM, streptomycin; TB, tuberculosis.

On admission, her temperature was 39.1°C, blood pressure was 70/53 mmHg, and respiration rate was 28 with a pulse rate of 136 beats per minute. Her blood oxygen saturation level using pulse oximetry was 96% under an oxygen flow of 10 L/min via nonrebreathing oxygen mask with a reservoir bag. Her Glasgow Coma Scale score was 14: eye opening, 4; verbal response, 5; and motor response, 5. Her height was 149 cm, and her body weight was 33.4 kg. Her body mass index (BMI) was 15.0. Physical examination revealed mild and coarse crackles in the right and left sides of the chest. Blood tests showed a leukocyte count: 1600/*μ*L, neutrophil count: 1208/*μ*L, lymphocyte count: 221/*μ*L, hemoglobin: 10.7 g/dL, platelet count: 154000/*μ*L, total bilirubin: 1.5 mg/dL, CRP: 0.59 mg/L, lactate dehydrogenase: 509 U/L, creatinine: 0.29 mg/dL, prothrombin time: 12.5 sec, activated partial thromboplastin time: 26.5 sec, fibrinogen: 85 mg/dL, D-dimer: 2.4 *μ*g/mL, Krebs von den Lungen-6 (KL‐6): 423.3 U/mL, pulmonary surfactant protein-D: 199 ng/mL, and brain natriuretic peptide (BNP): 42.8 pg/mL. The arterial blood gas analysis, while under an oxygen flow of 10 L/min via nonrebreathing oxygen mask with a reservoir bag, showed pH: 7.504, partial pressure of arterial oxygen (PaO_2_): 159.0 mmHg, partial pressure of arterial carbon dioxide (PaCO_2_): 33.4 mmHg, base excess: 3.2 mEq/L, HCO3: 26.3 mEq/L, and arterial oxygen saturation: 100%. Calculated fraction of inspiratory oxygen (FiO_2_) under an oxygen flow of 10 L/min via nonrebreathing oxygen mask with a reservoir bag was 0.76 based on the previous report [6]. The sequential organ failure assessment score was 5. Cytomegalovirus pp65 antigenemia was negative. Serum *β*-D glucan level was 286 pg/mL, and the T-SPOT-TB test was positive. Antigen tests for the presence of species of *Aspergillus*, *Candida*, and *Cryptococcus neoformans* were all negative. The PCR-based sputum test for *Pneumocystis jirovecii* DNA was positive. The antigen test for coronavirus disease 2019 was negative. *Streptococcus pneumoniae* and *Legionella pneumoniae* urinary antigen tests were negative. CT scan showed grand glass opacity in all fields with consolidation within the right and left lungs (Figure 2) and mildly swollen kidneys without hydronephrosis. The echocardiography was normal. Her PaO_2_/FiO_2_ was 209.2 mmHg. She was diagnosed as having septic shock [7] and also diagnosed as acute respiratory distress syndrome (ARDS) based on the Berlin definition [8]. The clinical course is shown in Figure 3. She was treated with 3 g of meropenem per day. Because of worsening respiratory condition, the dose of methylprednisolone was increased to 80 mg per day for her ARDS based on previous studies [9, 10]. These treatments made her afebrile; however, the respiratory condition gradually worsened, and she required high-flow nasal oxygen on day 3. On the same day, the blood and urinary culture were positive for extended-spectrum *β*-lactamase-producing *Escherichia coli*. Since the result of the sputum test for TB was positive on day 6, daily administration of antitubercular agents including, 200 mg of isoniazid, 500 mg of levofloxacin, and 500 mg of streptomycin was initiated. However, the hypoxemia gradually worsened, and she died of respiratory failure on day 7. With written permission from her family, an autopsy was performed.

### 2.1. Autopsy (Figure 4)

**Figure 4:**
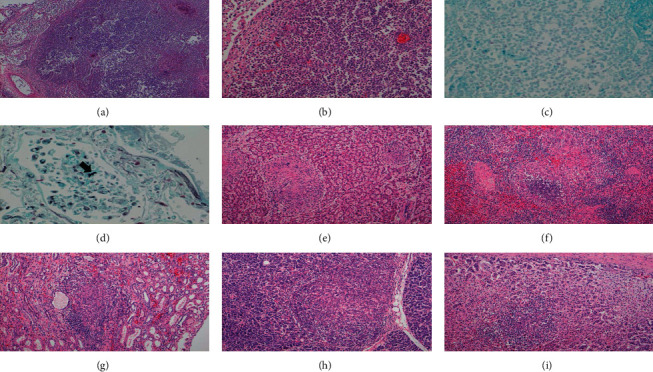
Excessive neutrophil accumulation with necrosis in the lung ((a) ×40; (b) ×100). Numerous acid-fast bacilli with Ziehl–Neelsen stain ((c) ×400) and round to oval cysts with Grocott's methenamine silver nitrate stain ((d) ×400). Granulomas present in the liver (e), spleen (f), kidney (g), pancreas (h), and adrenal gland (i).

Bilateral lungs showed gross necrotic and cystic lesions of a maximum size of 30 mm. Histologically, the necrotic lesions consisted infiltration of neutrophils, macrophages, and giant cells with severe inflammatory exudate. However, granulomatous formations were not observed. Ziehl–Neelsen staining aided the detection of acid-fast bacilli (Figure 4(a)–4(c)). These lesions seemed to be of primary TB. A few numbers of pneumocystis organisms were identified in the lungs using Grocott's methenamine silver nitrate staining (Figure 4(d)). Numerous granulomas were present as extrapulmonary lesions in the liver, spleen, kidneys, pancreas, and adrenal glands (Figures 4(e)–4(i)). Some of them contained caseous necrosis and/or Langhans giant cells. Mild lymphocyte infiltration was observed around the renal pelvis.

## 3. Discussion

We present a fatal case of RA with disseminated TB, PCP, and septic shock due to pyelonephritis caused by extended-spectrum *β*-lactamase-producing *Escherichia coli* treated with MTX, methylprednisolone, and TCZ.

To the best of our knowledge, there are no reports on patients with RA concurrently developing TB, PCP, and bacterial septic shock. In patients with HIV, Sheikholeslami et al. reported that 10% of the respiratory samples were coinfected with *Mycobacterium tuberculosis* and *Pneumocystis jirovecii* [11]. However, there is only one report on the development of coinfection with *Mycobacterium tuberculosis* and *Pneumocystis jirovecii* among patients with systemic rheumatic diseases [12]. Patients with RA develop infections more frequently than patients without RA do, and several reports have revealed that the use of biologicals increase infections, particularly pulmonary infections [13]. According to the mandatory postmarketing surveillance programs in Japan, reports have shown that 1.3%–2.2% of patients with RA treated with biologicals developed bacterial pneumonia, 0.18%–0.44% developed PCP, and 0.05%–0.28% developed TB [1–3]. In the case reported here, she did not receive prophylaxis for TB because she had no history of TB, and her T-SPOT-TB test and sputum test for *Mycobacterium tuberculosis* were negative. However, several investigators reported that older age, low BMI, immunosuppressive treatment, lymphocytopenia, and hypoalbuminemia are factors for the false-negative T-SPOT-Tb assay [14, 15]. In our patient, she was older age, had hypoalbuminemia, low BMI, and received immunosuppressive treatment, which might have suggested false-negative for the T-SPOT-Tb assay. According to the Japan College of Rheumatology, patients who are suspected to have latent TB include the history of TB, chest X-ray findings compatible with old TB, and positive interferon-gamma release assay or tuberculin test should receive prophylaxis if physicians administer biologics [16]. In our patient, chest CT before TCZ treatment showed thickening of the pleura and a nodule, suggesting that prophylaxis might have required for TB despite no history of TB, and the T-SPOT-TB test and sputum test for *Mycobacterium tuberculosis* were negative.

Alternatively, an age of at least 65 years, a daily dose of prednisolone of at least 6 mg, and the presence of coexisting pulmonary disease are the risk factors for PCP in patients with RA receiving infliximab; patients with two or three of these risk factors had a significantly higher cumulative probability of PCP than patients with no risk factors [17]. Our patient had two risk factors, including old age and glucocorticoid use, which showed a high risk for the development of PCP [17]. She received inhaled pentamidine every 4 weeks; however, she developed PCP. Schneider et al. reported that inhaled pentamidine once a month was less effective as primary prophylaxis against PCP than SMX/TMP in patients with HIV [18].

Biologics and glucocorticoids increase the risk of hospital-acquired infections [4]. In particular, glucocorticoids are associated with a dose-dependent increase in the risk of serious infections [19]. Our patient received 8 mg/day of methylprednisolone in combination with MTX and TCZ, which is consistent with previous studies [4, 19]. In addition, glucocorticoids decrease serum IgG levels, which is associated with severe bacterial infections [20, 21]. According to the Clinical Guidelines for Immunoglobulin Use, patients with secondary antibody deficiency with a serum IgG level <500 mg/dL should receive intravenous immunoglobulin therapy [22]. In our patient, the serum IgG level was 294 mg/dL on admission and 487 mg/dL on day 29; this showed secondary antibody deficiency. In addition, our patient received ADA treatment before TCZ. Anti-TNF inhibitors-experienced patients were also associated with significant serious infections and the incidence rate of TB after the initiation of TNF-*α* inhibitors was very high [23, 24]. On the other hand, the risk of infection with TCZ was similar to that with other biologicals [25], and TCZ does not influence on the IFN-*γ* synthesis by TB antigens [26]. However, TCZ masks clinical symptoms and decreases serum CRP levels [27], and early diagnosis of infection is very difficult in patients receiving TCZ.

Subesinghe et al. reported that serum lymphocyte nadir counts below 1000/*μ*L in patients with RA, particularly below 500/*μ*L, were at high risk of serious infections; this reduction appears within the immediate 30 days prior to severe infections [28]. In our patient, the serum lymphocyte counts on day 29 after TCZ was normal, although it decreased on day 44. On admission, she had a history of generalized fatigue with cough for two weeks. The serum lymphocyte counts increased after admission, despite the high-dose glucocorticoid therapy. These results show that serum lymphocytopenia preceded infection, and serum lymphocyte count might be a useful marker to help predicting opportunistic infections as well as the other severe infections.

In conclusion, patients with RA may develop severe opportunistic infections, in particular receiving biologics. We must be aware of the potential of opportunistic infections and may consider prophylaxis for opportunistic infections in these patients with high risk.

## Figures and Tables

**Table 1:** Laboratory tests before admission.

Day after TCZ	WBC (/*μ*L)	Lymphocyte count (/*μ*L)	CRP (mg/dL)	Albumin (g/dL)	IgG (mg/dL)
Day 1	12820	1615	5.4	2.3	686
Day 15	8770	1666	0.09	2.7	524
Day 29	5570	1688	0.01	2.8	487
Day 44	4610	724	0.01	3.3	N.A
Day 69 (on admission)	1600	221	0.59	3.0	294

CRP, C reactive protein; IgG, immunoglobulin G; N.A, not available; TCZ, tocilizumab; WBC, leukocyte count.
